# Percutaneous Image-Guided Non-Target Renal Biopsy in Cancer Patients: A Tertiary Cancer Center Experience

**DOI:** 10.3390/curroncol33040192

**Published:** 2026-03-30

**Authors:** Mohamed E. Abdelsalam, Milan N. Patel, Ryan D. Murray, Shahroz Khalid Aziz, Haley Shields, Pamela Chien, Steven Yevich, Zeyad A. Metwalli, Zhongya Wang, Jamie S. Lin, Steven Y. Huang, David Irwin, Thomas Lu, Stephen R. Lee, Ala Abudayyeh, Peiman Habibollahi, Bruno C. Odisio, Kamran Ahrar, Sanjay Gupta

**Affiliations:** 1 Department of Interventional Radiology, The University of Texas MD Anderson Cancer Center, Houston, TX 77030, USA; 2 Department of Radiology, Keck School of Medicine, University of South California, Los Angeles, CA 90033, USA; 3 Department of Radiology, The University of Texas McGovern Medical School, Houston, TX 77030, USA; 4 Department of Biostatistics, The University of Texas MD Anderson Cancer Center, Houston, TX 77030, USA; zwang28@mdanderson.org; 5 Department of Internal Medicine, Section of Nephrology, The University of Texas MD Anderson Cancer Center, Houston, TX 77030, USA

**Keywords:** non-target, biopsy, renal, cancer, nephropathy

## Abstract

**Objective**: To assess the safety and diagnostic outcomes of image-guided, non-target renal biopsies performed in cancer patients. **Materials and Methods**: We retrospectively identified patients who underwent percutaneous, image-guided, non-target renal biopsy between January 2017 and December 2020 in our institution. We recorded demographics, clinical, procedural, and pathologic details. Univariate and multivariable logistic regression models were used to assess the association between various variables and diagnostic yield or development of adverse events. **Results**: A total of 318 biopsies were performed in 318 patients (178 male, 140 female) with a median BMI of 28.4 kg/m^2^. Median systolic and diastolic BP at the time of biopsy were 133 mmHg and 74 mmHg, respectively. Tissue was obtained using 18-gauge needles (99%). Adverse events were documented in 57 cases (18%), with 12 cases (3.8%) classified as grade 2 or higher per SIR classification. Diagnosis was achieved in 310 biopsies (97%). The median number of the glomeruli identified by light microscopy, immunofluorescence, and electron microscopy was 25, 8, and 3, respectively, and a higher number of identified glomeruli was associated with diagnostic yield in univariate analysis, although not in multivariable analysis. Diastolic BP higher than 80 mmHg and CT imaging guidance were associated with the development of adverse events in univariate analysis, and CT use remained so in the final multivariable analysis (*p* < 0.001). No other variables, including pre-biopsy anticancer or immunotherapy medications, were associated with increased risk of adverse events. **Conclusions**: Percutaneous, image-guided, non-target renal biopsy in cancer patients using an 18-gauge needle has a high diagnostic yield and safety profile.

## Simple Summary

This study looked at how safe and effective it is to perform native kidney tissue (non-tumor) biopsy using imaging guidance in cancer patients. We reviewed records of cancer patients who had this type of kidney biopsy at our hospital between 2017 and 2020. We collected information about their health, the procedure, and the biopsy results. We also looked at what factors were linked to complications or successful diagnosis. A total of 318 patients (178 men and 140 women) had the biopsy done, and about four tissue samples were taken per person. Overall, 97% of biopsies provided enough tissue for diagnosis. Only 4% of patients had moderate or more serious side effects, and none had long-term issues. Higher diastolic blood pressure was linked to a higher risk of side effects. The number of kidney structures (glomeruli) seen under the microscope was linked to diagnostic success. In conclusion, this type of kidney biopsy is generally safe and gives reliable results in cancer patients. It carries a low risk of complications, especially when done with image guidance.

## 1. Introduction

Since its introduction in the 1950s [1], the non-target renal biopsy has become a fundamental component for the study of the intrinsic renal disease [2]. Tissue sampling enables pathologists to evaluate the structural and functional status of the renal parenchyma, establish a definitive diagnosis, and determine the extent and severity of underlying pathologic processes [2,3,4]. For instance, immune-related acute interstitial nephritis or thrombotic microangiopathy can be reliably diagnosed only through renal tissue evaluation. Likewise, in patients with chronic kidney disease of uncertain etiology, or those with proteinuria, hematuria, or rapidly rising creatinine, histologic assessment via biopsy plays a critical role in determining prognosis and guiding immunosuppressive therapy [2,3,4]. However, the diagnostic benefit of the biopsy is highly dependent on the adequacy and quality of the obtained specimen [5,6,7,8,9,10]. Several factors have been associated with improved adequacy of the tissue sample, including a larger needle [8], bigger tissue sample [10], automatic core biopsy system [11], immediate assessment by an on-site pathologist [12], and the incorporation of imaging guidance [5,9,13,14]. Real-time imaging guidance has been shown to enhance both the safety profile and diagnostic adequacy in percutaneous renal biopsy. Among various technical approaches, the cortical tangential trajectory has shown efficacy and has offered high diagnostic adequacy while minimizing the incidence of post-procedural adverse events. This technique has been validated under both ultrasound and computed tomography (CT) guidance [15,16,17,18].

The cancer patient population presents a unique clinical context: patients with malignancy often develop renal dysfunction secondary to complex, multifactorial causes, including nephrotoxic chemotherapeutic agents, immunotherapy-induced glomerulopathies, obstructive uropathy, paraneoplastic syndromes, or underlying comorbid conditions. In the context of precision oncology and individualized treatment strategies, especially molecularly targeted agents and immune checkpoint inhibitors, percutaneous non-target renal biopsy has become increasingly pivotal in guiding patient-specific clinical decision-making. There is a distinct paucity of data regarding procedural risk and diagnostic efficacy of non-target renal biopsies in cancer patients, who often present with thrombocytopenia, anemia, use of anticancer medications, or other comorbidities that could theoretically increase procedural risk.

To address this knowledge gap, we conducted a comprehensive retrospective review of non-target renal biopsies performed under image guidance in cancer patients at a large tertiary cancer center over a 4-year period. Our primary objective was to evaluate the procedural safety and diagnostic outcomes associated with percutaneous, image-guided, non-target renal biopsies in cancer patients, with a focus on the influence of different clinical or procedural variables on the diagnostic yield and safety profile of the procedure.

## 2. Materials and Methods

### 2.1. Study Design

This study was designed as a single-center, retrospective cohort analysis. We reviewed the institutional interventional radiology biopsy database to identify all adult patients with an existing diagnosis of malignancy who underwent percutaneous, image-guided, non-target renal biopsy between 1 January 2017 and 31 December 2020. Institutional Review Board approval was obtained prior to data collection, and a waiver of informed consent was granted due to the retrospective nature of the study. This study complied with the Health Insurance Portability and Accountability Act (HIPAA) regulations.

### 2.2. Patient Characteristics: Inclusion and Exclusion Criteria

Patients were eligible for inclusion in this study if they met the following criteria:

1. Age ≥ 18 years at the time of the procedure.
2. Histologically confirmed malignancy at the time of renal biopsy.
3. Underwent percutaneous, image-guided non-target renal biopsy (i.e., sampling the parenchyma, not a radiographically identified mass or focal lesion).

Biopsies performed for renal mass characterization were excluded.

### 2.3. Biopsy Technique

All biopsy referrals undergo a thorough clinical evaluation to assess appropriateness for image-guided biopsy. At our institution, percutaneous non-target renal biopsies are exclusively conducted by interventional radiologists using imaging guidance. Procedures are typically performed with the patient under moderate sedation, although general anesthesia may be employed in select cases, with continuous hemodynamic monitoring throughout the biopsy.

Pre-procedural optimization of coagulation parameters is standard practice. Specifically, a platelet count > 50 × 10^9^/L and an international normalized ratio (INR) < 1.5 are prerequisites for proceeding with the biopsy. In accordance with our institutional guidelines, antiplatelets are discontinued for a minimum of 5 days, anticoagulants are suspended for at least 2 days prior to the procedure, and low-molecular-weight heparin (enoxaparin) is suspended for 1 day prior to the procedure.

Both ultrasonography and CT are considered image-guidance modalities, with the choice determined at the operator’s discretion. A coaxial technique is used for tissue acquisition. Under image guidance, while the patient is lying prone, a 17-gauge coaxial introducer is advanced just beyond the renal capsule. Through this access, an 18-gauge core biopsy needle is used to harvest tissue using a cortical tangential trajectory, aiming to maximize diagnostic yield while minimizing procedural risk.

Our standard practice is to acquire four core specimens per procedure without on-site immediate adequacy assessment. The samples are submitted for histopathological evaluation at a centralized pathology laboratory with subspecialty expertise in native renal pathology.

### 2.4. Data Collection

Electronic medical records and images were reviewed to collect demographic, clinical, procedural, and histopathologic data. The following variables were extracted:

- Demographics and Clinical Data: Age, sex, body mass index (BMI), blood pressure (systolic and diastolic) at time of procedure, serum creatinine (mg/dL), estimated glomerular filtration rate (eGFR; mL/min/1.73 m^2^), INR, platelet count (×10^9^/L), and use of anticoagulant or antiplatelet medications.
- Oncologic Therapy: Status and type of systemic therapy (cytotoxic chemotherapy, targeted therapy, immunotherapy, or combination therapy) at the time of biopsy.
- Procedural Parameters: Imaging guidance modality (operator dependent, either ultrasound, CT, or both), performing interventional radiologist, laterality of biopsy (right or left kidney), depth of renal cortex from skin surface (cm), needle gauge used (operator dependent, either 18-gauge or 20-gauge), and number of cores obtained.
- Adverse events: All post-biopsy adverse events recorded and graded according to the Society of Interventional Radiology (SIR) Classification System [19]. Adverse events of grade 2 or higher were categorized as clinically significant.
- Histopathology and Diagnostic Yield: All specimens were processed by renal pathologists with expertise in intrinsic renal diseases. Histopathologic evaluation included light microscopy (LM), immunofluorescence (IF), and electron microscopy (EM). The number of glomeruli identified using each modality was recorded. A biopsy was considered diagnostically adequate if the renal pathologist was able to render a definitive or clinically actionable diagnosis based on available tissue.

### 2.5. Outcome Measures

The primary objective of the study was to assess (1) diagnostic yield, defined as the proportion of biopsies resulting in a histopathologic diagnosis; and (2) Adverse events, categorized by severity per SIR criteria. The secondary objective was to assess associations between patient- or procedure-related variables and the occurrence of diagnostic yield or procedural adverse events.

### 2.6. Statistical Analysis

Continuous variables were summarized using medians and ranges, and categorical variables were reported as frequencies and percentages. Univariate and multivariable logistic regression models were used to examine potential associations between different clinical or procedural variables (e.g., imaging modality, blood pressure, renal depth) and (a) diagnostic yield and (b) complication occurrence. A *p*-value of <0.05 was considered statistically significant. SAS version 9.4. Software was used for all statistical analyses.

## 3. Results

### 3.1. Patient and Clinical Characteristics

Between January 2017 and December 2020, a total of 318 percutaneous, image-guided, non-target renal biopsies were performed by 25 interventional radiologists in 318 oncology patients at our tertiary care cancer center. All biopsies were conducted in native kidneys; none were anatomically abnormal or transplanted kidneys. Of the total cohort, 178 patients (56%) were male, and 140 (44%) were female, with a median age of 64 years (range 19–89 years). The median BMI was 28.4 kg/m^2^, ranging from 15.7 to 51.8 kg/m^2^. The median laboratory parameters at the time of biopsy included platelet count 194 × 10^9^/L (range 45–380 × 10^9^/L), INR 1.04 (range 0.80–1.87), serum creatinine 2.30 mg/dL (range 0.52–7.8), and eGFR 28.0 mL/min/1.73 m^2^ (range 15–151 mL/min/1.73 m^2^). Sixty-three patients (20%) were receiving active anticancer therapy or immunotherapy at the time of the biopsy. Demographic and clinical characteristics are listed in Table 1.

### 3.2. Procedural Variables

**Figure 1:**
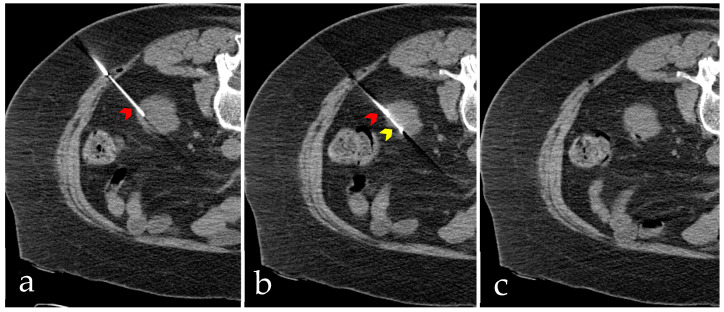
Example of a percutaneous non-target renal biopsy performed under computed tomography (CT) guidance. A 61-year-old man with a known diagnosis of lymphoma developed new-onset proteinuria and was subsequently referred to interventional radiology for the biopsy. The patient had a body mass index of 37.5 kg/m^2^, and the measured distance from the skin surface to the renal parenchyma was 8.5 cm. (**a**) Axial non-contrast CT image showing a 17-gauge coaxial introducer (red arrowhead). (**b**) Axial non-contrast CT image showing an 18-gauge core biopsy needle (yellow arrowhead) through the coaxial introducer (red arrowhead) utilizing a cortical tangential approach to sample the renal cortex. A total of four core tissue samples were obtained. (**c**) Post-procedural non-contrast CT image showing no radiologic evidence of hemorrhagic complication.

The median systolic and diastolic blood pressure readings at the time of biopsy were 133 mmHg (range 89–200 mmHg) and 74 mmHg (range 46–106 mmHg), respectively. All biopsies were performed using a coaxial needle approach under image guidance: ultrasonography in 188 cases (59%), CT in 127 cases (40%), and a combination of both in three cases (1%). The median depth from the skin to the renal cortex was 6.4 cm (range 2.0–15.5 cm). Core tissue acquisition was achieved using an 18-gauge needle (99%) predominantly, with a 20-gauge needle used in only three cases (1%), and a median of four cores was obtained (range 3–6). Figure 1 illustrates a representative case in which the biopsy was performed under CT guidance.

### 3.3. Diagnostic Yield and Histopathologic Outcomes

Tissue diagnosis was achieved in 310 biopsies (97%). The median number of the glomeruli identified using LM, IF, and EM was 25 (range 0–77), 8 (range 0–45), and 3 (range 0–14), respectively. The median number of arteries identified by LM was two (range 0–11). Univariate analysis showed that a higher number of arteries and glomeruli identified was associated with a higher likelihood of establishing a definitive diagnosis. However, this association was not retained in the multivariable analysis, which did not reveal a statistically significant relationship between these parameters and diagnostic outcome. BMI, sex, depth of renal parenchyma from the skin surface, imaging guidance modality, performing interventional radiologist, needle gauge, number of tissue samples obtained, indication for the biopsy, and primary malignancy were not associated with the diagnostic yield. Table 2 summarizes patient characteristics based on diagnostic status

### 3.4. Adverse Events

Procedural adverse events were documented in 57 cases (18%), with 12 cases (3.8%) classified as grade 2 or higher per SIR classification [19]. Among these 12 patients, 9 underwent blood transfusion, including 2 who required embolization, and 3 patients needed escalation of care for enhanced monitoring or pain management; summarized in Table 3. Both diastolic blood pressure higher than 80 mmHg (*p* = 0.003) and the use of CT for image guidance (*p* < 0.001) were associated with the development of adverse events in the univariate analysis. In the multivariable model, only CT as the imaging modality remained significantly associated with the development of adverse events after adjusting for diastolic blood pressure (*p* < 0.001), whereas diastolic blood pressure exceeding 80 mmHg showed a borderline association with the development of adverse events after adjusting for the imaging modality used (*p* = 0.059). Sex, years of experience of the interventional radiologist, BMI, size of needle, number of cores obtained, creatinine, eGFR, indication for the biopsy, primary malignancy, presence of anemia (or its severity), and prebiopsy anticancer immunotherapy, chemotherapy, antiplatelet or anticoagulation medications were not associated with the risk of adverse events (Table 4, Table 5 and Table 6).

## 4. Discussion

In recent years, at our institution, the advent and widespread adoption of immunotherapy as a cornerstone of oncologic treatment have further amplified the clinical utility of non-target renal biopsies. Despite this clinical importance, non-target renal biopsies may be underused in oncological patients, partly due to concerns over bleeding risks and diagnostic uncertainty. By systematically analyzing a large institutional cohort, we sought to provide much-needed data to inform procedural planning and risk stratification when considering non-target renal biopsy in cancer patients. Our results show that non-target renal biopsy in cancer patients using 18-gauge needles has a high success rate in terms of diagnostic yield and a low complication rate, thereby supporting its benefit and safety in this unique clinical context.

Currently, there is no universally accepted definition of sample adequacy for non-target renal biopsies in native kidneys. However, in the context of non-target biopsies, where the goal is to evaluate intrinsic parenchymal disease rather than to characterize a focal lesion, obtaining a representative sampling of glomeruli, tubules, and vasculature is essential to achieve a diagnosis. In the current study, adequacy was defined pragmatically based on the primary objective of the biopsy, i.e., whether a definitive histopathologic diagnosis was achieved or not. With a median glomerular count of 25 in LM analysis, 8 in IF analysis, and 3 in EM analysis, a diagnostic yield of 97% was reported in our study. This rate is consistent with, and in some cases exceeds, that of prior studies involving renal biopsies in non-cancer populations. Previous literature has reported diagnostic adequacy ranging from 89% to 100% for native kidney biopsies, with variability in the sizes of needles used for sampling [20,21,22,23,24].

In a study conducted by Carrington et al. [20], involving a cohort of 192 patients who underwent non-target renal biopsy, diagnostic tissue adequacy was achieved in 97% of cases using a 14-gauge core biopsy needle. In another study by Liu et al. [17], 431 patients underwent CT-guided native renal biopsies, most of which were performed using an 18-gauge needle, and the ability to establish a definitive histopathologic diagnosis was achieved in 97.7% of cases [17]. We found no significant associations between patient or procedural characteristics and diagnostic yield. Patient BMI, renal depth, imaging modality used, number of cores obtained, needle gauge, type of malignancy, and indication for the biopsy were not associated with the ability to obtain a diagnostic specimen. This underscores the reliability of the coaxial 18-gauge approach across a wide range of clinical scenarios, including patients with high BMI.

Our data also confirm that non-target renal biopsy is a safe procedure, with an overall adverse events rate of 18%, and most adverse events were self-limited (SIR grade 1) and required no therapeutic intervention. The rate of clinically significant adverse events (SIR grade ≥ 2) was 3.7%, in line with previously published complication rates for renal biopsy [22,25,26,27,28]. In a study by Korbet et al. [22] involving 1055 non-target renal biopsies, the authors reported a major complication rate of 6.6%, with blood transfusions required in 5.3% of cases. In the current study, two factors were associated with increased complication risk: elevated diastolic blood pressure (>80 mmHg) and use of CT guidance during biopsy. Hypertension is a well-established risk factor for post-biopsy hemorrhage [29,30] and has been included in pre-procedural risk assessment [4]. Our findings reinforce the importance of pre-biopsy blood pressure optimization, particularly in cancer patients, who may have other risk factors increasing the risk of bleeding. The higher complication rate observed with CT-guided procedures may be multifactorial. Although CT offers precise anatomic delineation, it lacks real-time needle visualization, which may theoretically increase the risk of non-target injury and hemorrhage. Also, it may be that patients with more complicated anatomy or poor visualization under ultrasonography were chosen for CT imaging guidance. However, the observed association may also simply reflect the modality’s superior sensitivity for detecting immediate post-biopsy bleeding, thereby acting as a potential confounder.

The strengths of our study include its relatively large cohort size, standardized biopsy technique, and evaluation within a single institution with subspecialty pathology review. This minimizes variability and enhances internal validity. The inclusion of 25 radiologists also supports the generalizability of our findings across a diverse operator experience spectrum.

We acknowledge the limitations of our study, including the retrospective design, which may introduce selection bias. Although adverse events were systematically graded using SIR criteria, minor events may have been underreported if they were not explicitly documented in the electronic medical record or if patients sought medical care at an outside institution. Additionally, our findings reflect practice in a high-volume cancer center and may not be fully generalizable to non-oncologic populations or lower-resource settings.

In conclusion, our study showed that percutaneous, image-guided, non-target renal biopsy in cancer patients using an 18-gauge needle has a high diagnostic yield and is associated with minimal morbidity and no long-term adverse events, suggesting that this technique is broadly effective across a wide range of clinical renal disease presentations in this patient population. Future prospective studies incorporating multivariable risk models are warranted to further refine patient selection, procedural planning, and risk stratification.

## Figures and Tables

**Table 1:** Summary of patients, clinical and procedural characteristics—all patients.

Variable		All (*n* = 318)
Age:		
	Median:	64 years
	Range:	19–89 years
Sex		
	Female	140 (44%)
	Male	178 (56%)
Indication for biopsy, *n* (%)		
1. Adult Nephrotic Syndrome		3 (1%)
2. Proteinuria		47 (15%)
3. Progressive increase in serum creatinine with microscopic evidence of casts		3 (1%)
4. Systemic disease (immunologic or paraneoplastic) with suspected renal involvement		2 (1%)
5. Impaired renal function of unclear etiology, e.g., drug-induced nephritis		165 (52%)
6. Examine the severity of damage or the progression of an already-known kidney disease		98 (31%)
Primary malignancy		
	Hematologic	121 (38%)
	Non-hematologic	185 (58%)
	Both	12 (4%)
BMI, kg/m^2^		
	Median	28.4
	Range	(15.7–51.8)
Creatinine, mg/dL		
	Median	2.30
	Range	(0.52–50.00)
eGFR, mL/min/1.73 m^2^		
	Median	28.0
	Range	(1.8–151.0)
INR		
	Median	1.04
	Range	(0.80–1.87)
Pre-procedure blood pressure:		
Systolic, mmHg	Median	133
	Range	(89–200)
	>140	125 (39)
	≤140	193 (61)
Diastolic, mmHg	Median	74
	Range	(46–106)
	>80	91 (29)
	≤80	227 (71)
Years of experience as an interventional radiologist, *n* (%)		
	0–5 years	97 (31%)
	5.1–10 years	45 (14%)
	10.1–15 years	93 (29%)
	15.1–20 years	45 (14%)
	20.1–25 years	28 (9%)
	25.1–30 years	10 (3%)
Depth of the kidney, cm		
	Median	6.4
	Range	(2.0–15.5)
Imaging modality		
	US	188 (59%)
	CT	127 (40%)
	US and CT	3 (1%)
Size of needle		
	18 gauge	315 (99%)
	20 gauge	3 (1%)
No. of cores obtained		
	Median	4
	Range	(3–6)
No. of arteries by LM		
	Median	2
	Range	(0–11)
No. of glomeruli by LM		
	Median	25
	Range	(0–77)
No. of glomeruli by IF		
	Median	8
	Range	(0–45)
No. of glomeruli by EM		
	Median	3
	Range	(0–14)
Diagnostic status		
	Yes	310(97%)
	No	8 (3%)
Adverse events		
	Yes	57 (18%)
	No	261 (82%)
Patients with antiplatelet or anticoagulation medication		
	Yes	99 (31%)
	no	219(69%)
Patients with anticancer/immunotherapy medication (*n* = 63)		
	ICI PD-L1	31 (48)
	Chemotherapy	6 (10)
	TKI	23 (37)
	MEK inhibitor	5 (8)
	Other	10 (16)
Number of medication categories per patient:		
	Median	1
	Range	1–3

BMI, body mass index; eGFR, estimated glomerular filtration rate; INR, international normalized ratio; US, ultrasonography; CT, computed tomography; LM, light microscopy; IF, immunofluorescence; EM, electron microscopy; ICI, immune checkpoint inhibitors; TKI, tyrosine kinase inhibitor.

**Table 2:** Summary of comparisons of characteristics based on diagnostic status.

Variable		Diagnostic		*p*-Value
		Yes (*n* = 310)	No (*n* = 8)	
Indication, *n* (%)		*n* (%)	*n* (%)	1.00
	1	3 (1)	0 (0)	
	2	46 (15)	1 (12.5)	
	3	3 (1)	0 (0)	
	4	2 (1)	0 (0)	
	5	160 (53)	5 (62.5)	
	6	96 (31)	2 (25)	
Primary malignancy, *n*				0.38
	H	116 (37)	5 (63)	
	NH	182 (58)	3 (38)	
	Both	12 (4)	0	
Pre-procedure BP: systolic, mmHg				0.24
	Median	133	126.5	
	Range	(89–200)	(106–158)	
	>140	123 (40)	2 (25)	0.49
	≤140	187 (60)	6 (75)	
Pre-procedure BP: diastolic				0.70
	Median	74	71.5	
	Range	(46–106)	(59–88)	
	>80	91 (29)	0 (0)	1.00
	≤80	219(71)	8 (100)	
Years of experience as an interventional radiologist, *n* (%)				0.11
	0–5 years	95 (31)	2 (25)	
	5.1–10 years	42 (14)	3 (37.5)	
	10.1–15 years	92 (30)	1 (12.5)	
	15.1–20 years	45 (15)	0 (0)	
	20.1–25 years	27 (9)	1 (12.5)	
	25.1–30 years	9 (3)	1 (12.5)	
No. of arteries by LM				0.023
	Median	2	0	
	Range	(0–11)	(0–5)	
No. of glomeruli by LM				0.003
	Median	25	0	
	Range	(0–77)	(0–60)	
No. of glomeruli by IF				0.047
	Median	8	1	
	Range	(0–45)	(0–11)	
No. of glomeruli by EM				0.013
	Median	3	0	
	Range	(0–14)	(0–1)	

LM, light microscopy; IF, immunofluorescence; EM, electron microscopy; H, hematological malignancy; NH, non-hematological malignancy; BP, blood pressure.

**Table 3:** Summary of patients’ adverse events, SIR category, and management.

Adverse Event (AE)	Escalation of Care	SIR AE Category
Moderate hematoma	Admission 2 days after the procedure	2
Chest pain and dyspnea	Admission; medically treated; no bleeding identified on imaging; may have been related to anesthesia	2
Large hematoma	Admission; severe pain 2 weeks post-procedure; large subcapsular hematoma; blood transfusion; possible delayed rupture of a pseudoaneurysm	2
Large hematoma	Admission; blood transfusion; angiography; embolization	2
Hematuria	Admission for pain control; Foley for hematuria; blood transfusion	2
Pain	Admission; underwent multiple US and CT over the subsequent days following biopsy, with no abnormalities identified on imaging	2
Large hematoma	Admission; blood transfusion	2
Large hematoma	Admission; blood transfusion	2
Large hematoma	Admission; blood transfusion	2
Moderate hematoma	Admission; blood transfusion	2
Large hematoma	Admission; blood transfusion	2
Hematuria	Blood transfusion; angiography; embolization	2

**Table 4:** Summary of patients’ characteristics by the presence of adverse events.

Variable		Adverse Events		*p*-Value
		Yes (*n* = 57)	No (*n* = 261)	
Sex		*n* (%)	*n* (%)	0.10
	Female	31 (54)	109 (42)	
	Male	26 (46)	152 (58)	
Indication for biopsy				0.16
	1	0 (0)	3 (1)	
	2	5 (9)	42 (16)	
	3	0 (0)	3 (1)	
	4	0 (0)	2 (1)	
	5	26 (46)	139 (53)	
	6	26 (46)	72 (28)	
Primary malignancy				0.38
	H	25 (44)	96 (37)	
	NH	30 (52)	155 (60)	
	Both	2 (4)	10 (13)	
BMI				0.21
	Median	29.2	28.2	
	Range	(17.8–51.8)	(15.7–49.2)	
Creatinine				0.79
	Median	2.30	2.30	
	Range	(1.11–21.00)	(0.52–50.00)	
eGFR				0.59
	Median	25.0	28.0	
	Range	(3.4–80.0)	(1.8–151.0)	
INR				0.20
	Median	1.02	1.04	
	Range	(0.90–1.42)	(0.80–1.87)	
Antiplatelet/anticoagulation medications				0.64
	Yes	16 (28)	83 (32)	
	No	41 (72)	178 (68)	
Pre-procedure BP: systolic, mmHg				0.29
	Median	134	133	
	Range	(97–200)	(89–196)	
	>140	24 (42)	101 (39)	0.66
	≤140	33 (58)	160 (61)	
Pre-procedure BP: diastolic, mmHg				0.010
	Median	78	74	
	Range	(54–101)	(46–106)	
	≤80	31 (54)	196 (75)	0.003
	>80	26 (46)	65 (25)	
Pre-procedure anemia				0.43
	Yes	50 (88)	215 (82)	
	No	7 (12)	46 (18)	
Years of experience as an IR MD, *n* (%)				0.62
	0–5 years	14 (25)	83 (32)	
	5.1–10 years	9 (16)	36 (14)	
	10.1–15 years	16 (28)	77 (30)	
	15.1–20 years	12 (21)	33 (13)	
	20.1–25 years	5 (9)	23 (9)	
	25.1–30 years	1 (2)	9 (3)	
Depth of kidney (cm)				0.14
	Median	6.9	6.3	
	Range	(3.1–15.5)	(2.0–15.3)	
Imaging modality				<0.001
	US	17 (30)	171 (66)	
	CT	40 (70)	87 (33)	
	US/CT	0 (0)	3 (1)	
Size of needle				0.08
	18 gauge	55 (97)	260 (100)	
	20 gauge	2 (4)	1 (<1)	
No. of cores obtained				0.35
	Median	4	3.5	
	Range	(3–6)	(2–6)	
No. of arteries by LM				0.96
	Median	2	2	
	Range	(0–7)	(0–11)	
No. of glomeruli by LM				0.93
	Median	22	25	
	Range	(0–68)	(0–77)	
No. of glomeruli by IF				0.95
	Median	7	8	
	Range	(0–24)	(0–45)	
No. of glomeruli by EM				0.69
	Median	3	3	
	Range	(0–10)	(0–14)	

BMI, body mass index; eGFR, estimated glomerular filtration rate; INR, international normalized ratio; US, ultrasonography; CT, computed tomography; LM, light microscopy; IF, immunofluorescence; EM, electron microscopy; H, hematological malignancy; NH, non-hematological malignancy; BP, blood pressure; IR MD, Interventional Radiologist.

**Table 5:** Summary of comparison of patients’ and procedural characteristics by the presence of major adverse events.

Measure	Level	Significant Adverse Events		*p*-Value
		No (*N* = 306)	Yes (*N* = 12)	
Primary malignancy, *n* (%)				0.26
	H	113 (38)	4 (33)	
	NH	176 (60)	7 (58)	
	H, NH	5 (2)	1 (8)	
Years of experience as an IR MD, *n* (%)				0.022
	0–5 years	95 (31)	2 (17)	
	5.1–10 years	40 (13)	5 (42)	
	10.1–15 years	93 (30)	0 (0)	
	15.1–20 years	42 (14)	3 (25)	
	20.1–25 years	27 (9)	1 (8)	
	25.1–30 years	9 (3)	1 (8)	
Indication, *n* (%)				0.15
	1	3 (1)	0 (0)	
	2	47 (15)	0 (0)	
	3	3 (1)	0 (0)	
	4	2 (1)	0 (0)	
	5	161 (53)	4 (33)	
	6	90 (29)	8 (67)	
Imaging modality				0.15
	US	184 (60)	4 (33)	
	CT	119 (39)	8 (67)	
	US/CT	3 (1)	0 (0)	
Pre-procedure IR H&P BP: diastolic				0.24
	Median	74	78.5	
	Range	(46–106)	(60–101)	
Sex				0.77
	Female	134 (44)	6 (50)	
	Male	172 (56)	6 (50)	
Size of needle				0.11
	18	304 (99)	11 (92)	
	20	2 (1)	1 (8)	

**Table 6:** Summary of comparison of characteristics based on diagnostic status and adverse events in patients with anticancer/immunotherapy medication at the time of biopsy (*n* = 63).

Medication Category	Diagnostic		
	Yes (*n* = 62)		No (*n* = 1)
	*n* (%)	*n* (%)	*p*-value
ICI PD-L1	30 (48)	1 (100)	0.49
Chemotherapy	6 (10)	0 (0)	1.00
TKI	23 (37)	0 (0)	1.00
MEK inhibitor	5 (8)	0 (0)	1.00
Other	10 (16)	0 (0)	1.00
Number of categorized medications:			0.65
Median	1	1	
Range	(1–3)	(1–1)	
	Adverse Events		
	Yes (*n* = 8)		No (*n* = 55)
	*n* (%)	*n* (%)	*p*-value
ICI PD-L1	3 (38)	28 (51)	0.71
Chemotherapy	1 (12)	5 (9)	0.57
TKI	3 (38)	20 (36)	1.00
MEK inhibitor	1 (12)	4 (7)	0.51
Other	3 (38)	7 (13)	0.11
Number of categorized medications:			0.46
Median	1	1	
Range	(1–3)	(1–2)	

ICI, immune checkpoint inhibitor; PD-L1, programmed death-ligand 1; TKI, tyrosine kinase inhibitor; MEK inhibitor, mitogen-activated protein kinase inhibitor.

## Data Availability

The original contributions presented in this study are included in the article. Further inquiries can be directed to the corresponding author.
